# Improving the Room-Temperature Ferromagnetism in ZnO and Low-Doped ZnO:Ag Films Using GLAD Sputtering

**DOI:** 10.3390/ma14185337

**Published:** 2021-09-16

**Authors:** Marcio A. Correa, Armando Ferreira, Raphael M. Tromer, Leonardo D. Machado, Matheus Gamino, Sergio A. N. França Junior, Felipe Bohn, Filipe Vaz

**Affiliations:** 1 Departamento de Física, Universidade Federal do Rio Grande do Norte, Natal 59078-900, RN, Brazil; marciocorrea@fisica.ufrn.br (M.A.C.); leodantasm@gmail.com (L.D.M.); mgamino@fisica.ufrn.br (M.G.); sergiofrancajn@gmail.com (S.A.N.F.J.); felipebohn@fisica.ufrn.br (F.B.); 2 Centro de Física, Universidade do Minho, 4710-057 Braga, Portugal; armando.f@fisica.uminho.pt; 3 Departamento de Física Aplicada, Universidade Estadual de Campinas, Campinas 13083-970, SP, Brazil; tromer@fisica.ufrn.br; 4 Center for Computational Engineering and Sciences, Universidade Estadual de Campinas, Campinas 13083-970, SP, Brazil

**Keywords:** zinc oxide, thin films, Glancing Angle Deposition, doping, biosensors, sputtering

## Abstract

ZnO and doped ZnO films with non-ferromagnetic metal have been widely used as biosensor elements. In these studies, the electrochemical measurements are explored, though the electrical impedance of the system. In this sense, the ferromagnetic properties of the material can be used for multifunctionalization of the sensor element using external magnetic fields during the measurements. Within this context, we investigate the room-temperature ferromagnetism in pure ZnO and Ag-doped ZnO films presenting zigzag-like columnar geometry. Specifically, we focus on the films’ structural and quasi-static magnetic properties and disclose that they evolve with the doping of low-Ag concentrations and the columnar geometry employed during the deposition. The magnetic characterization reveals ferromagnetic behavior at room temperature for all studied samples, including the pure ZnO one. By considering computational simulations, we address the origin of ferromagnetism in ZnO and Ag-doped ZnO and interpret our results in terms of the Zn vacancy dynamics, its substitution by an Ag atom in the site, and the influence of the columnar geometry on the magnetic properties of the films. Our findings bring to light an exciting way to induce/explore the room-temperature ferromagnetism of a non-ferromagnetic metal-doped semiconductor as a promising candidate for biosensor applications.

## 1. Introduction

ZnO and doped ZnO:NFM materials, with NFM being a non-ferromagnetic metal such as Cu or Ag, have received increasing attention in the last decade, not just for their interesting electric properties [1,2,3,4,5,6,7,8], but also due to their ferromagnetic features. With concern to the photocatalytic applications [1] ZnO:NFM has been used to suppress electron-hole recombinations, improving the efficiency of sensor applications. From the electrical properties side, ZnO films have a strong dependence with $O_{2}$ gas flow in reactive magnetron sputtering. The variation of $O_{2}$ gas flow allows us to modify the electrical nature of ZnO films, from metallic to insulator, going through a semiconductor phase [2]. Consequently, ZnO and ZnO:NFM can present an interesting appeal for temperature sensors.

Remarkably, such materials may exhibit room-temperature ferromagnetism (RTFM) [9,10,11,12]. This is associated with so-called bound magnetic polarons (BMPs), mediated with shallow donor electrons, or structural defects and dopants substitution [13,14,15]. In particular, for pure ZnO films, oxygen and zinc vacancies can be present, contributing to the RTFM. However, these mechanisms seem to be strongly dependent on the used deposition process. Considering the element Ag as a non-magnetic metallic dopant, numerous studies have explored the electrical and magnetic properties of ZnO:NFM for distinct sample geometries [5,6,12]. For instance, Ali et al. [16] investigated Ag-doped ZnO thin films with variable Ag concentration. In this case, the RTFM was interpreted in terms of the substitution of Ag in the Zn sites of the ZnO materials. More recently, Qi et al. [17] explored the photocatalytic and antibacterial performance of transition metal-doped ZnO nanoparticles. Specifically, the authors performed the doping of a ZnO matrix with Mn, Fe, Co, Ni, and Cu. Then, the density functional theory (DFT) was used to disclose the photocatalytic mechanism responsible for the observed behavior.

Regarding technological applications, ZnO and doped ZnO films have received special attention associated with biosensors [18,19,20,21,22]. Between the distinct ways to employ these systems in biosensors, the electrochemical impedance is the most widely used [23]. From a general point of view, the efficiency of the sensor element is strongly dependent on structural, electrical, and, more recently, magnetic properties. This last property, in particular, can be explored as an interesting functionality in future devices. The electrical impedance of ferromagnetic materials can be easily manipulated through an external magnetic field. Since we are able to modify the magnetic permeability of the materials changing the impedance response of the system, it is a well-known magnetoimpedance effect [24,25].

Surprisingly rare studies consider the columnar structure’s influence on the magnetic response of ZnO and doped ZnO:NFM materials. In this sense, the employment of reactive magnetron sputtering associated with Glancing Angle Deposition (GLAD) emerges as an interesting experimental mechanism to produce a system with controllable columnar structure [26]. The GLAD technique allows us to control the inclination of the columnar growth, as well as produce films with zigzag-like geometry. The last, in particular, brings advantages of control, not just the structural properties of the studied films, but also to modify/control their electrical and magnetic properties [27,28,29], essential properties to optimize the response of future sensor elements.

Looking in this direction, our group has recently shown the RTFM in ZnO:Ag-doped system with high Ag concentrations has been considered. In particular, we explored the magnetic properties of ZnO:Ag films in a wide range of temperatures and magnetic fields, revealing that structural columnar growth influences the RTFM verified in this set of samples [30]. However, high concentration of Ag on the ZnO site brings some difficulties with respect to theoretical analysis, mainly when density functional theory (DFT) is considered. Theoretical study using DFT bringing to light the mechanisms yielding modifications in the magnetic properties of undoped and doped ZnO materials have been performed in our study, a procedure of paramount importance to the comprehension of the fundamental physics behind such magnetic behavior.

Within this context, DFT calculations appear to be a powerful tool to study not just the electric and structural properties of ZnO and ZnO:NFM materials, but also the mechanisms responsible for the RTFM behavior [31,32,33].

From a general perspective, these studies explore the multifunctionality of ZnO and doped ZnO:NFM materials for future biosensor applications [20,21,22,34,35,36]. In particular, Zhu and co-workers [20] presented a systematic study of ZnO-based nanostructures for biosensor applications. For instance, in this article, the authors showed the efficiency of ZnO-based nanostructures as enzymatic, hydrogen peroxide, and glucose biosensors. Moreover, the response of ZnO films as an antibacterial agent is discussed. In another interesting study proposed by Tak et al. [22] Ni-doped ZnO films were successfully employed to compose a biosensor DNA to detect a life-threatening disease, meningitis.

The comprehension of the mechanism that leads to the RTFM can help us to produce systems with tunable electric and magnetic properties by modifying the columnar structure of the films.

In this work, we explore the RTFM in undoped ZnO and doped ZnO:Ag films with a low concentration of Ag and zigzag-like columnar structure. For this purpose, we realize a systematic study of structural and quasi-static magnetic properties of the films with 0, 2, and 4 wt.% doping of Ag on ZnO films. The structural behavior shows a clear modification of the space group of the system as Ag concentration increases, although the zigzag-like structure remains unchanged. Furthermore, the magnetic characterization reveals ferromagnetic behavior at room temperature for all studied samples, for the system without Ag doping. By considering computational simulations, we investigate the origin of ferromagnetism in ZnO and Ag-doped ZnO and interpret our results in terms of the Zn vacancy dynamics and its substitution by an Ag atom in the site and columnar structure employed during the deposition. Our findings provide the exciting ability of ZnO films and their capacity to compose multifunctional systems.

## 2. Experiment

**Figure 1:**
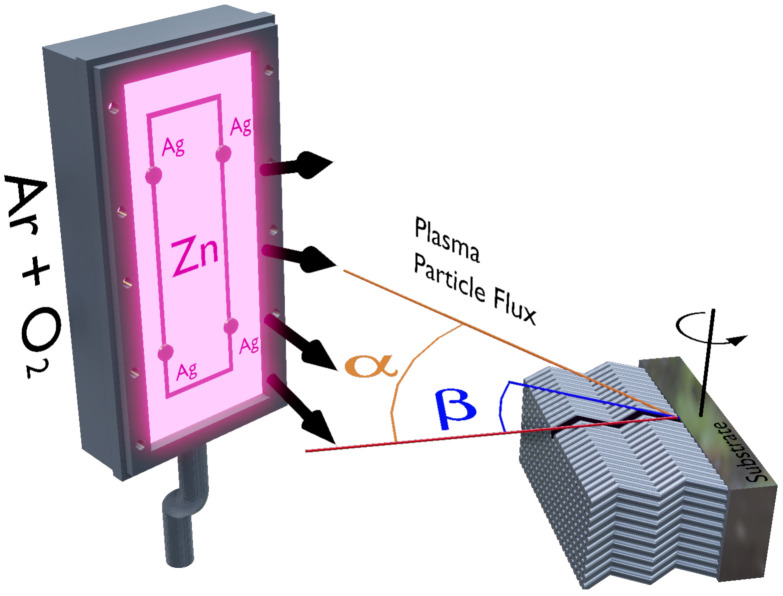
Schematic representation of the GLAD sputtering in which the α and β angles are indicated. The Zn target was decorated with a specific amount of Ag pellets (dark cylinders), symmetrically distributed along the main erosion area (50 cm${}^{2}$) represented through the dark rectangle in the ZnO target.

We investigated here a set of samples composed of pure ZnO and doped ZnO:Ag films doped with distinct Ag concentrations. Specifically, the doped ZnO:Ag films were prepared with the low-Ag concentrations of 2% and 4%, from now on known as ZnO:2Ag and ZnO:4Ag, respectively. The samples were produced onto glass substrate with dimensions of 4×4 mm${}^{2}$ using reactive magnetron sputtering and GLAD techniques, simultaneously. The depositions were performed through a metallic zinc target with 99.96 at.% purity and dimensions of 20×10×0.6 cm${}^{3}$ connected to a DC source. To produce the doped ZnO:Ag films, the Zn target was decorated with a specific amount of Ag pellets, symmetrically distributed along the main erosion area (50 cm${}^{2}$), in order to set the silver concentration at 2 wt.% and 4 wt.% [30,37]. In particular, the zigzag-like columnar structure was obtained by rotating the substrate in relation to the plasma particle flux (α) using the so-called tangent and cosine rules [38,39,40]. Here, we use fixed $\alpha =60^{\circ }$ for around 8 minutes for each step (4 columns here), which allow us to reach columns with $\beta \approx 45^{\circ }$ with respect to the normal direction of the substrate, as depicted in Figure 1. The depositions were carried out with base pressure of $6\times 10^{-7}$ Torr, deposition pressure of $5\times 10^{-3}$ Torr with Ar at 25 sccm and O${}_{2}$ at 16 sccm constant flows. The Zn target was connected to a DC source in which was set at 377 V. The structural properties of the films were investigated by X-ray diffraction (XRD) experiments performed using a Rigaku Miniflex II diffractometer with Cu${}_{\alpha }$ radiation ($\lambda_{K\alpha }=1.54060$ Å) in a θ−2θ configuration. The morphology of the films were observed using a FEB/SEM microscopy, NanoSEM-FEI Nova 200 system. In particular, the microscopy images were observed from a top-view (not shown here) and cross-section perspective. This last enables us to verify the zigzag-like induced structure during the deposition. The room-temperature magnetic behavior was investigated through magnetization curves acquired using a Quantum Design Dynacool Physical Property Measurement System (PPMS) with the vibrating sample magnetometer (VSM) module. The magnetization curves were acquired with the magnetic field of ±2.0 T applied in the film plane. However, to turn better visualization of the ferromagnetic contribution in our systems, here we present the magnetic results in a limited magnetic field range of ±0.2 T. It is worth mentioning that we removed from the results the diamagnetic contribution of the sample holder as well as the ZnO matrix contribution, by employing a careful protocol [30]. In particular, the first measurement is realized with the sample holder without the sample, while the ZnO matrix contribution is obtained by discounting the linear contribution of the ZnO and ZnO:Ag curves. In this sense, the magnetization curves discussed in the following fundamentally bring the ferromagnetic signal of pure ZnO and doped ZnO:Ag films.

## 3. Computational Simulation

We used the SIESTA software [41] to perform DFT simulations and investigate the origin of ferromagnetism in pure ZnO and doped ZnO:Ag films. In our calculations, we considered a ZnO supercell system composed of 108 atoms. The calculations were performed within the generalized gradient approximation (GGA) using the PBE functional [42] to describe the exchange-correlation term. We used a double-zeta plus polarization (DZP) basis set [41]. The core electrons were also not considered explicitly, and the norm-conserving Troullier–Martins pseudopotential with Bylander factorized form [43,44] was employed. The energy mesh cut-off was set to 2720 eV assuming that the Brillouin zone is modeled by *k* points along with the hexagonal cell, and the reciprocal space was sampled considering the Monkhorst–Pack scheme with a 2×2×2 k-point mesh [45]. Regarding the convergence of the self-consistency cycle, we assumed that it was achieved when the maximum difference between the density matrix elements was smaller than $10^{-4}$ eV. Finally, during our geometry optimization calculations, both ionic positions and lattice vectors were varied, and optimization continued until the force on each atom was smaller than 0.05 eV/Å. In all calculations, we considered spin polarization. We also determined the density of states (DOS) for a spin-up and spin-down electrons for pure ZnO, for ZnO with a Zn vacancy, and Ag-doped ZnO. Asymmetries in the DOS for spin-up and -down electrons occur when the magnetic moment is non-zero.

## 4. Results and Discussion

**Figure 2:**
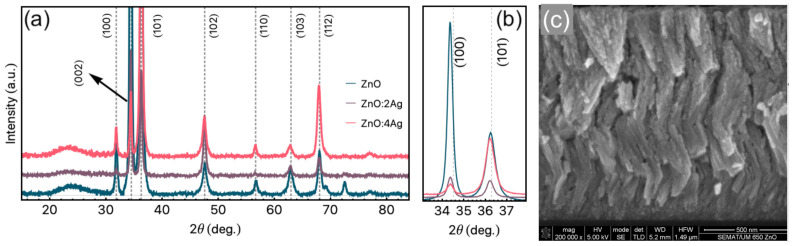
(**a**) X-ray diffraction results obtained for our samples. The peaks were indexed using the ICSD-01-072-0067 card (space group p63mc). In particular, the vertical axis is linear scale. (**b**) view of the X-ray diffraction showing the peaks related to the preferential growth direction. (**c**) Representative cross-section SEM image. Here we show the cross-section image obtained for the pure ZnO film. Using the software ImageJ we infer the thickness of the film as around 1.2μm.

Figure 2a shows the XRD diffraction results for the ZnO and ZnO:Ag films. We verify a hexagonal structure for the ZnO, identified through the presence of peaks associated with (100), (002), and (110) ZnO planes (space group P63mc). Although peaks related to Ag are not found in the XRD spectrums due to the low Ag concentration, the modification on the structural behavior due to the increase of Ag concentration is remarkable. From the results, we observe a clear modification of the preferential growth direction of the films. This feature is depicted in Figure 2b, in which an amplified view in the 2θ range of $33^{\circ }$ up to $38^{\circ }$ is presented. In this case, we observe a change of the preferential growth direction, starting from (100) preferential growth, for the undoped ZnO film, to (101) preferential direction for the ZnO:Ag films. This behavior is associated with the modification in the space group of the ZnO:Ag films [30]. Although we have performed Rutherford Backscattering Spectroscopy (RBS) to verify the Ag content, the results are inconclusive due to the low concentration of the dopant. However, our structural findings agree with previous work for similar experimental procedures [46,47].

Regarding SEM measurements, Figure 2c shows a representative cross-section image taken for the pure ZnO film. We infer the thickness of the samples of around 1.2μm for all samples studied in this work. In particular, the zigzag-like columnar growth is evident due to the deposition process employed in the sample preparation. This columnar structure is found for all studied samples, i.e., pure ZnO and doped ZnO:Ag films. Although the Ag doping modifies the preferential growth direction, this procedure does not significantly alter the columnar growth. It is important to emphasize that the zigzag-like columnar structure enables us to improve the RTFM in the ZnO and doped ZnO:Ag films [30]. This feature can be connected with the defects inserted due to the controlled and periodic breaks in the columnar growth.

**Figure 3:**
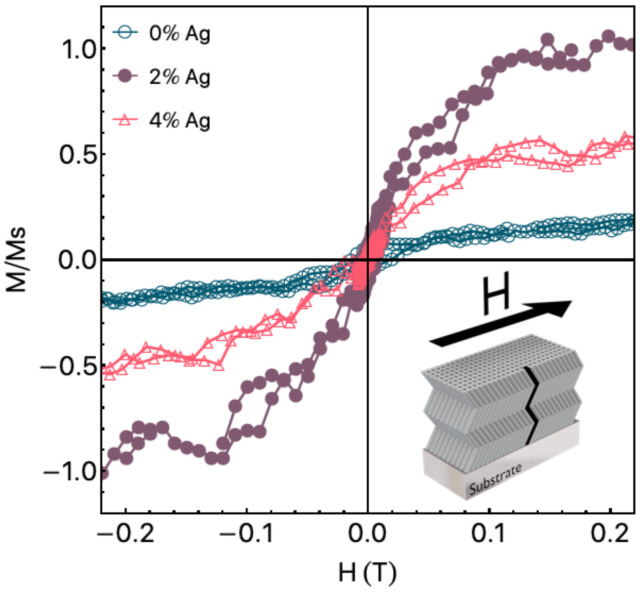
Normalized magnetization curves for the ZnO film and the Ag-doped ZnO films. The magnetization curves are normalized by the saturation magnetization obtained for the ZnO:2Ag film. The inset depicts the field applied direction during the magnetization measurements.

Figure 3 shows the in film plane normalized magnetization curves. As aforementioned, we removed from the curves the diamagnetic contributions of the sample holder and ZnO matrix. By employing this procedure, we can observe the paramagnetic/ferromagnetic contribution of the ZnO and ZnO:Ag films. It is important to point out that the films present isotropic magnetic behavior in the film plane. The magnetization curves present hysteretic behavior, with coercive field H${}_{c}$ values of 16.4 mT, 5.1 mT, and 2.2 mT for the pure ZnO film, ZnO:2Ag, and ZnO:4Ag, respectively. To make a direct comparison of the magnetic properties for distinct samples easier, we normalized the magnetization curves by the saturation magnetization of the ZnO:2Ag film, which by the way has the highest M${}_{s}$ value for this set of samples. Although the calculation of M${}_{s}$ for thin films is an arduous task, here we observe a considerable M${}_{s}$ value of around 0.78 emu/cm${}^{3}$ for the pure ZnO film. This result follows previous results for a similar studied system [30]. These remarkable results can be associated with two distinct mechanisms. The first one due to the Zn vacancies associated with the deposition process. The second one is due to the zigzag-like columnar geometry employed through GLAD sputtering.This procedure leads to a broken of the columnar growth generating defects on the ZnO structure, increasing the saturation magnetization. Our findings disclose to previous studies in which the sputtering technique was used to deposit undoped ZnO film. In general, the undoped ZnO films do not present any RTFM contribution [15,16]. However, they are in accordance with others in which mechanisms associated with shallow donor electrons are considered, generating bound magnetic polarons leading to a ferromagnetic contribution [13]. With the insertion of a low Ag concentration, we observe an evolution of the saturation magnetization value, which increases abruptly for the ZnO:2Ag film, and decreases in the following for the ZnO:4Ag. Remarkably, the saturation magnetization of the ZnO:4Ag film is roughly half of the one found for the ZnO:2Ag film. Similar results may be found in the literature for ZnO doped with non-ferromagnetic metals. For instance, Ali et al. [15] uncovered the RTFM of Cu-doped ZnO films with Cu concentrations similar to that explored here. In the study, the authors observed that the saturation magnetization increases abruptly for the ZnO film doped with 2% of Cu, followed by a decrease when the ZnO is doped by 4% of Cu. However, they do not verify any ferromagnetic contribution for the undoped ZnO film, distinctly from our results.

From a technological perspective, the present results allow us to produce ZnO and doped ZnO:Ag films in which we infer a ferromagnetic response on the studied system, in addition to the well-known electrical properties. Although the induced magnetic moment is small, our findings bring to light an exciting rote to infer a ferromagnetic response on the ZnO system that can be easily manipulated during electrochemical measurements, modifying the electrical impedance by employing an external magnetic field.

**Figure 4:**
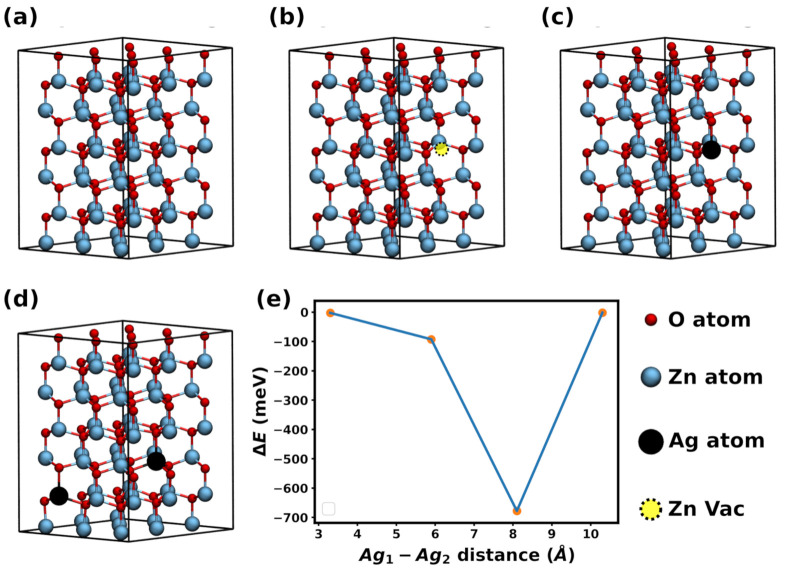
ZnO structures investigated here. Specifically, the sketches show the supercell used in the simulation for the (**a**) pure; (**b**) one Zn vacancy; (**c**) one Ag dopant; (**d**) two Ag dopants systems. We studied four structures with two Ag dopants, and the other three are omitted. The distance between Ag atoms differs in these structures. (**e**) The energy difference between ferromagnetic and antiferromagnetic configurations for the four ZnO structures containing two Ag atoms.

From a theoretical perspective, the mechanisms responsible for the change in the magnetic properties are discussed in the following, in which we make use of DFT calculations to obtain the magnetic moment of Ag-doped supercell. Figure 4a shows the ZnO supercell with 108 atoms used in our simulations. After optimization, we obtained unit cell vectors a=b=3.30 Å and c=5.31 Å. To investigate the effects of Ag dopants on the magnetization, we also considered a supercell containing one silver atom, as we can see in Figure 4c, and four supercells containing two silver atoms, for which a representative example is depicted in Figure 4d. Furthermore, for comparison purposes, we also considered a structure containing a Zn vacancy, presented in Figure 4b.

Previous studies have demonstrated that Zn vacancies induce magnetic moment in ZnO [15,48]. Our calculations revealed non-zero magnetic moment in Ag-doped ZnO, in agreement with our experimental results previously discussed. Specifically, we obtained μ=5.35 emu/cm${}^{3}$ for the structure with one Ag atom, and μ=6.38 emu/cm${}^{3}$ for all structures with two Ag atoms.

**Figure 5:**
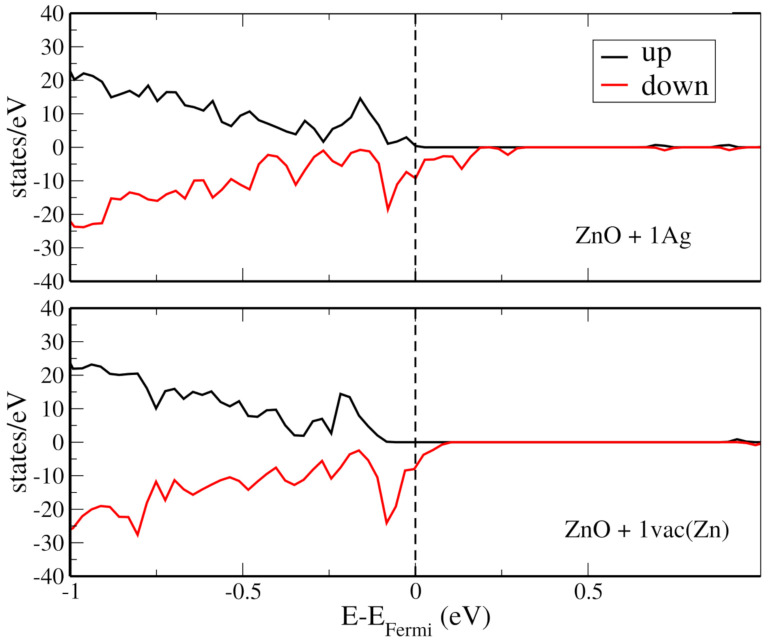
The the spin-polarized density of states for selected ZnO structures. On the top, spin-polarized density observed for a ZnO+1Ag system. On the bottom, the similar plot for the ZnO+1vac(Zn), i. e., a ZnO system with Zn vacancy.

Next, for each structure containing two Ag atoms, we calculated the energy difference between the ferromagnetic and antiferromagnetic configurations (ΔE), to evaluate the magnetic ordering stability. These calculations are presented in Figure 4e, in which the *x*-axis indicates the distance between the dopant atoms. It is interesting to notice that two configurations are likely to retain their magnetic moment at room temperature, i.e., large ΔE, but that this is unlikely to occur for the other two, |ΔE|<3 meV. Please note that we did not perform calculations at non-zero temperatures, and these observations arise from comparisons between |ΔE| and kT at room temperature, which is ∼0.026 eV. It is also curious to compare the magnetic moment imparted by Ag dopants and by a Zn vacancy. For the latter, we obtained μ=6.47 emu/cm${}^{3}$. Hence, for the considered structures, we found that a single Zn vacancy induces the same magnetic moment as two Ag atoms. In Figure 5, we show the total density of states for the ZnO doped with an Ag atom and with a Zn vacancy. For both cases, observe that the DOS for up and down spins is asymmetric, as it should be for a structure with non-zero magnetic moment. Furthermore, note that the Ag-doped structure presents states near the Fermi energy.

These simulation results allow us to interpret the fact that in the experiments, magnetization increases with the raise of Ag concentration at first and then decreases. Qualitatively, it is likely that for low Ag concentrations, the added silver atoms contributed to the magnetization. On the other hand, for higher concentrations, the added Ag atoms may have (*i*) reduced the concentration of Zn vacancies and/or (*ii*) interacted with nearby Ag atoms, possibly forming arrangements with low thermal stability. It is important to point out that the increase in Ag concentration to 10% or 20%, as addressed in Ref. [30], leads to remarkable modifications in this behavior. For higher values of Ag concentration, clusters of this material arise in the film, changing the Zn vacancy substitution. This effect leads to an increase of the magnetic moment [30].

As a final note, let us discuss how defects introduced by GLAD sputtering contribute to the improved RTFM in the investigated samples, specifically Zn vacancies. Towards this goal, we remark that the effect of increasing the concentration of Zn vacancies in a ZnO thin film has been investigated before [48]. The authors reported that the total magnetic moment more than doubled when the concentration of Zn vacancies doubled (the specific number depended on the position of the vacancies within the ZnO thin film). In addition, they found that the energy difference between ferromagnetic and antiferromagnetic couplings of the Zn vacancies was larger than the room-temperature thermal energy for all investigated structures (suggesting that the ferromagnetic ordering might be stable). Hence, the additional Zn vacancies generated through the use of the GLAD method should couple ferromagnetically, contributing to the increased saturation magnetization value of the ZnO films.

In a general point of view, the presented results reveal an interesting rote to induce the RTFM in ZnO and doped ZnO systems. This characteristic is associated with the well-known capability of the ZnO to compose biosensors, gas sensors, humidity sensors, among others, and allow us to produce multifunctional systems that can be activated by external magnetic fields, for instance.

## 5. Conclusions

In summary, here we have explored room-temperature ferromagnetism in pure ZnO and doped ZnO:Ag films produced by GLAD sputtering. Here we have shown the dependence of the structural and quasi-static magnetic properties of the films produced with zigzag-like columnar structure and low-Ag concentrations.

The structural characterization has demonstrated a hexagonal structure with a remarkable modification in the preferential growth direction when low Ag concentrations have been inserted as the dopant. The cross-section SEM images have proved the zigzag-like structure induced during the deposition due to the GLAD sputtering technique. The magnetic characterization has revealed ferromagnetic behavior at room temperature for all samples of our set. By considering computational simulations, we have addressed the origin of ferromagnetism in ZnO and Ag-doped ZnO and interpreted our results in terms of the Zn vacancy dynamics and its substitution of an Ag atom in the site. In particular, the RTFM observed for our pure ZnO film, without the Ag dopant, results from the interplay of the Zn vacancy and the structural defects associated with the columnar growth in a zigzag-like structure. However, the modification in the saturation magnetization of the doped ZnO films is a consequence of the Zn vacancy dynamics and its substitution by an Ag atom in the site. Here we integrate the experimental and theoretical results and explain the mechanism responsible for the RTFM induction in ZnO films doped with low NFM materials. Our findings provide the exciting ability of ZnO films and their capacity to compose multifunctional systems. In particular, magnetic behavior can be associated with the well-known gas sensing ability to compose smart sensors activated by external magnetic fields in the future.

## Data Availability

The data presented in this study are available on request from the corresponding author.
